# Nonlinear Optical Properties in an Epitaxial YbFe_2_O_4_ Film Probed by Second Harmonic and Terahertz Generation

**DOI:** 10.3390/ma16051989

**Published:** 2023-02-28

**Authors:** Hongwu Yu, Yoichi Okimoto, Atsuya Morita, Shuhei Shimanuki, Kou Takubo, Tadahiko Ishikawa, Shin-ya Koshihara, Ryusei Minakami, Hirotake Itoh, Shinichiro Iwai, Naoshi Ikeda, Takumi Sakagami, Mayu Nozaki, Tatsuo Fujii

**Affiliations:** 1 Department of Chemistry, Tokyo Institute of Technology, 2-12-1, Meguro, Tokyo 152-8551, Japan; 2 Department of Physics, Tohoku University, Sendai 980-8578, Japan; 3 Department of Physics, Okayama University, 3-1-1, Tsushimanaka, Okayama 700-8530, Japan; 4 Department of Applied Chemistry, Okayama University, 3-1-1, Tsushimanaka, Okayama 700-8530, Japan

**Keywords:** ferroelectrics, nonlinear optics, second harmonic generation, strongly correlated systems, thin film

## Abstract

An epitaxial film of YbFe_2_O_4_, a candidate for oxide electronic ferroelectrics, was fabricated on yttrium-stabilized zirconia (YSZ) substrate by magnetron sputtering technique. For the film, second harmonic generation (SHG), and a terahertz radiation signal were observed at room temperature, confirming a polar structure of the film. The azimuth angle dependence of SHG shows four leaves-like profiles and is almost identical to that in a bulk single crystal. Based on tensor analyses of the SHG profiles, we could reveal the polarization structure and the relationship between the film structure of YbFe_2_O_4_ and the crystal axes of the YSZ substrate. The observed terahertz pulse showed anisotropic polarization dependence consistent with the SHG measurement, and the intensity of the emitted terahertz pulse reached about 9.2% of that emitted from ZnTe, a typical nonlinear crystal, implying that YbFe_2_O_4_ can be applied as a terahertz wave generator in which the direction of the electric field can be easily switched.

## 1. Introduction

Ferroelectrics are a class of materials in which crystals have a spontaneous polarization that an external electric field can control. With this characteristic feature, ferroelectrics have been applied in various industries, such as information storage devices, capacitors, actuators, and nonlinear crystals. Particularly for memory devices, it is desirable to achieve ferroelectrics that operate at ultra-high speeds and conserve energy. For that purpose, several studies on ultrafast control in organic and inorganic ferroelectrics have been conducted [1,2,3]. Also, attention has been paid to developing novel ferroelectrics that operate at higher speeds and lower energies than conventional ferroelectrics.

To achieve these goals, the concept of “electronic ferroelectrics” has been proposed experimentally [4,5,6] and theoretically [7,8] since the beginning of the 21st century. The characteristics of electronic ferroelectrics are that the order of ions with different valances originates polarization and the direction of polarization can be reversed by electron transfer between those ions. While in conventional ferroelectrics, spontaneous displacement of the constituent atoms in real space from their equilibrium points is important to reverse polarization. Therefore, electronic ferroelectrics are expected to have a significantly faster response time of polarization reversal and a smaller coercive electric field than conventional ferroelectrics. Research on developing electronic ferroelectrics is attracting attention in materials science and ferroelectric applications owing to their unique properties such as low coercive field, excellent durability, and ultrafast response.

**Figure 1:**
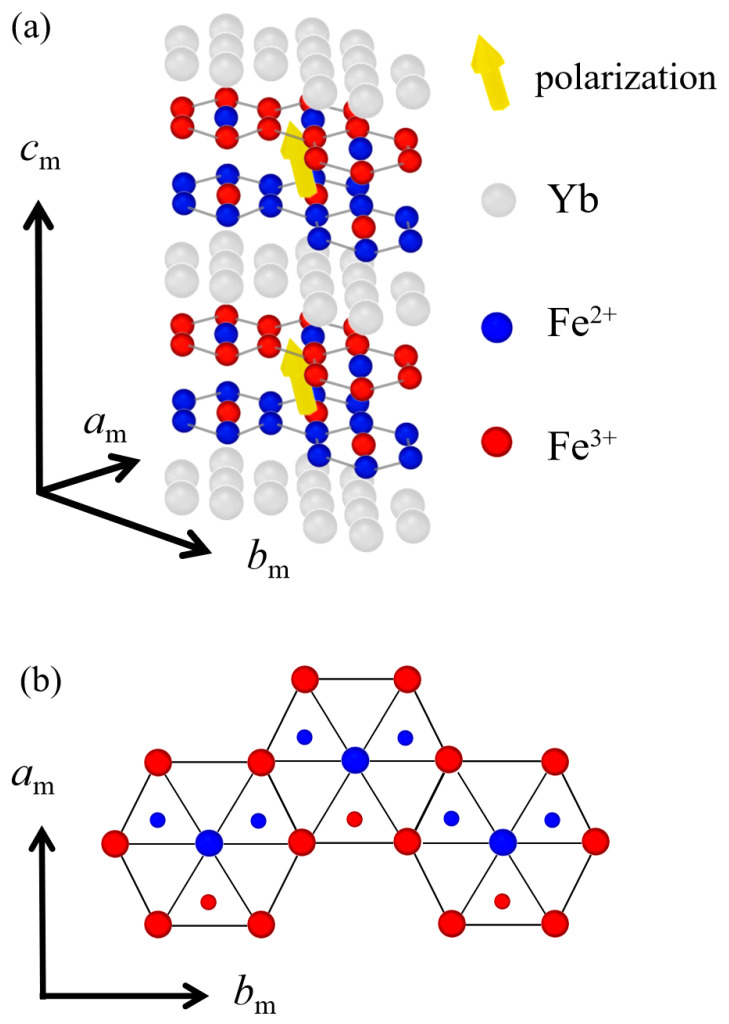
(**a**) Crystal structure of YbFe_2_O_4_ after Ref. [9]. Oxygens are not depicted for clarity. The yellow arrows denote the polarization direction. (**b**) Schematic view of the *W*-layer in a plane perpendicular to the *c_m_* axis. Red and blue circles denote Fe^3+^ and Fe^2+^, respectively and small ones Fe^3+^ and Fe^2+^ in the lower layer.

While looking for such electronic ferroelectrics, Ikeda et al. proposed an iron oxide complex, *R*Fe_2_O_4_ (*R* = rare earth ions), as a novel electronic ferroelectric material [4]. Figure 1a shows the crystal structure of YbFe_2_O_4_ without oxygen ions reported in a previous study [9]. This crystal is composed of an alternate stacking of the *R-*layer and *W*-layer along the *c*_m_ direction, as depicted in the schematic image. The *R-*layer comprises Yb^3+^ and oxygen ions, and the *W*-layer comprises two layers composed of Fe (Fe^2+^ and Fe^3+^) and oxygen ions stacked along the *c*_m_-axis direction [4,9]. Fe^2+^, Fe^3+^, and oxygen ions form a triangular lattice in the *W*-layer, as shown in Figure 1b, and this crystal has inversion symmetry (*R*$\bar{3}$*m*) above 500 K [10]. Furthermore, Fe^2+^ and Fe^3+^ in the *W*-layer show charge ordering with a three-fold periodic superstructure below 500 K [4]. Under these circumstances, the total amount of Fe^2+^ and Fe^3+^ is not equal in the upper and lower layers of the *W*-layer; In the upper layer, the number of Fe^3+^ is twice as many as that of Fe^2+^ and vice versa in the lower layer. This indicates the total charge of the upper layer is greater than that of the lower layer. Such charge disproportionation within the *W-*layer results in electric polarization in the direction of the yellow arrow in Figure 1a. Thus, polarization by electronic ordering occurs in this system at room temperature.

Although there have been several opinions regarding the presence or absence of polar structure in *R*Fe_2_O_4_ [11,12], recent measurements of second harmonic generation (SHG) in chemically equivalent *R* = Yb crystals have revealed the presence of electrical polarization [9]. In addition, analysis of the azimuthal dependence of SHG has shown that YbFe_2_O_4_ crystals are polarized and belong to the monoclinic *C_m_* point group. In this symmetry, the electric polarization exists in the *a_m_c_m_* plane, as shown in Figure 1a, supporting that the electric polarization forms owing to electron disproportionation in the upper and lower sheets of the *W*-layers, as proposed by Ikeda et al. [4].

To apply electronic ferroelectricity observed in a bulk YbFe_2_O_4_ to future ferroelectric devices, thin film crystals have been fabricated [13,14,15,16,17]. Several studies have succeeded in the epitaxial growth of YbFe_2_O_4_ thin films on various substrates, including yttrium-stabilized zirconia (YSZ) [13,14,15], MgO [16], and α-Al_2_O_3_ [17,18], whose structures are compatible with the hexagonal lattice of *R*Fe_2_O_4_. Furthermore, based on transmission electron microscopy (TEM) observations, Fujii et al. [14,17] first discovered that the resulting YbFe_2_O_4_ films have a superlattice structure owing to the order of Fe ions having the same three-fold periodicity as single crystals [4,19]. Thus, thin film crystals are expected to exhibit a polar structure like a single crystal. However, the X-ray diffraction analyses of *R*Fe_2_O_4_ have not determined the crystal symmetry consistent with the TEM measurement. Hence, it is yet to be determined whether these thin film crystals are polar or not.

In this report, we investigated two types of second-order nonlinear effects (SHG and terahertz generation) on a film of YbFe_2_O_4_ with a three-fold Fe ordering. The former experiment revealed the symmetry of the polarized film structure and the relationship between the polar direction and the crystal axes of the substrate. The latter demonstrated that YbFe_2_O_4_ film radiates a strong terahertz pulse owing to the polar structure, proposing a possible application of electronic ferroelectric oxide for nonlinear optical devices.

## 2. Experiment

We synthesized YbFe_2_O_4_ thin film crystal on YSZ (111) single crystal substrate. The films were deposited using the RF magnetron sputtering; the details are described elsewhere [14]. Table 1 shows the O_2_ partial pressure during sputtering and the retention time before deposition in which the substrate temperature is kept at ~1400 K. Conventional X-ray diffraction (XRD) and TEM measurements were performed to determine the crystallinity of the film. The *c_m_*-axis lattice constant obtained from XRD and the film thickness are also shown in Table 1.

**Figure 2:**
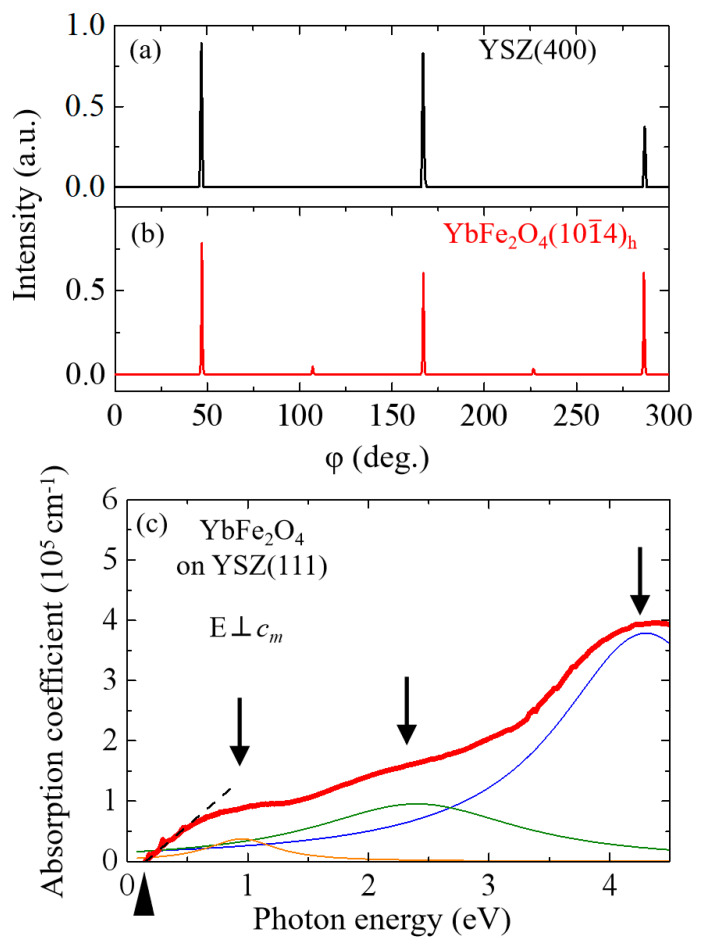
(**a**) Azimuth angle dependence of the (400) Bragg reflection for YSZ substrate. (**b**) Azimuth angle dependence of the (10$\bar{1}$4)_h_ Bragg reflection for YbFe_2_O_4_ thin film. (**c**) In-plane absorption coefficient spectrum of YbFe_2_O_4_ thin film.

Figure 2b shows the azimuth angle dependence of the (10$\bar{1}$4)_h_ Bragg reflection for the film, which shows the in-plane crystal orientation. For comparison, Figure 2a shows the azimuth angle dependence of the Bragg peak for the YSZ crystal, the substrate used for the film deposition. The appearance of the peaks at every 120° rotation of the crystal shows that the crystal has a three-fold symmetric structure. Like the YSZ substrate, the film shows a three-fold symmetry, although residues of a six-fold peak are observed owing to the crystalline domains (60° domains). The ratio of the six-fold to the three-fold component is about 8%, which is smaller than that observed in other *R*Fe_2_O_4_ films reported so far [14,17], indicating a good crystallization of YbFe_2_O_4_ film.

The normal incident transmission spectrum (*T*(ω)) was measured using a Fourier transform interferometer in the mid- and near-infrared region (0.01–1.0 eV). Above the near-infrared energy region (0.9–3.3 eV), we used grating-type monochromators and detected the transmitted monochromatic light with InGaAs (0.7–1.4 eV) and Si photodiodes (1.2–3.1 eV). The absorption coefficient spectrum (α(ω)) was calculated by the relation that α(ω) = −ln [*T*(ω)]/*l*, *l* being the thickness of the film.

**Figure 3:**
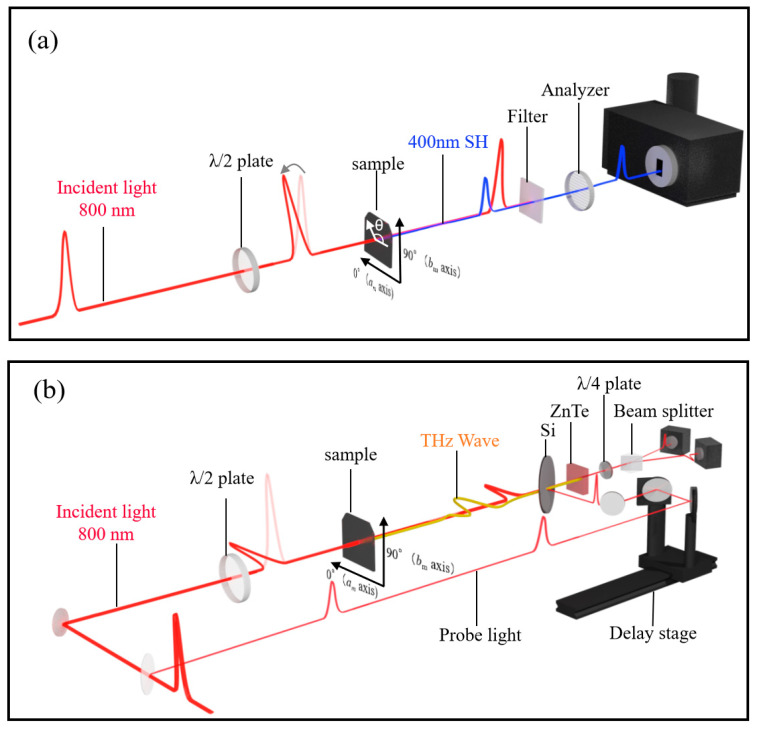
Schematic figures of the measurement systems of SHG (**a**) and terahertz radiation (**b**).

SHG and terahertz radiation were measured using a regenerate amplified mode-locked Ti: a sapphire laser source (pulse width: 35 fs; repetition rate: 1 kHz; center wavelength: 800 nm). Both measurement systems are depicted in Figure 3a,b. The thin film was irradiated with fundamental 800 nm pulses in normal incidence to avoid SHG or terahertz waves generated by the surface. The polarization of the 800 nm pulses to generate SHG and terahertz pulse is controlled using a half-wavelength plate. In the SHG measurement, an SHG pulse of approximately 400 nm was detected with a photomultiplier tube after removing the strong fundamental light using a high-pass filter and a grating-type monochromator. The terahertz pulse radiating in the direction of the *a_m_* axis of the film was detected with a conventional electro-optical sampling method. We carefully confirmed that neither SHG nor terahertz signal was observed by irradiating the YSZ substrate with the amplified fundamental pulses.

## 3. Results and Discussion

The absorption spectrum was measured to observe the electronic structure of the fabricated thin film. In Figure 2c, we show α(ω) of the film by a black line. As shown by the arrows in the figure, α(ω) contains three broad absorption bands at approximately 4.0 eV, 2.3 eV and 1.0 eV, which are assigned to the O 2*p* → Fe 3*d* charge-transfer transition, the Fe^2+^ on site transition, and the Fe^2+^ → Fe^3+^ *d*–*d* transition, respectively [15,18,20,21,22]. (In the figure, those absorption components extracted by Lorentz fitting are displayed by thin lines.)

An important feature in the electronic structure of the YbFe_2_O_4_ film has a larger spectral weight in the mid-infrared region than that of the single crystal of LuFe_2_O_4_ [22] or YbFe_2_O_4_ [23]. This discrepancy may be caused by the lattice mismatch between the film and the YSZ substrate [14]. This makes the electronic structure different from that in a single crystal of YbFe_2_O_4_, resulting in a reduction in the *d–d* transition energy and an increase in the spectral weight in the mid-infrared region. We extrapolated the rising part of the α(ω) of the thin film crystal as a dashed line and estimated the optical gap to be about 0.1 eV from its intersection with the abscissa (see the closed triangle), which is smaller than that of LuFe_2_O_4_ single crystal [22].

**Figure 4:**
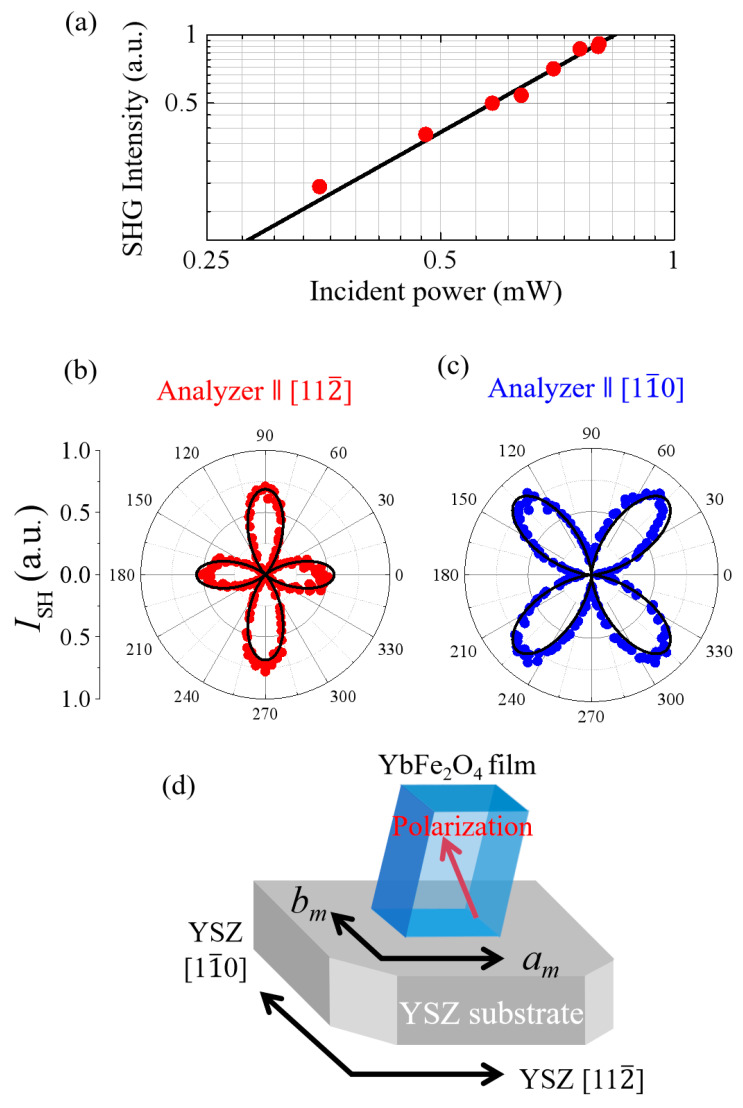
(**a**) Incident light intensity dependence of SHG emitted from YbFe_2_O_4_ thin film on the logarithmic scale. The solid lines are the fitting results (see text). (**b**,**c**) Azimuth angle dependence of SHG parallel (red circles) and perpendicular (blue circles) to the [11$\bar{2}$] direction of YSZ substrate. The solid lines are the fitted results based on the *C_m_* symmetry (see text). (**d**): A schematic of monoclinic unit cell of YbFe_2_O_4_ thin film fabricated on the YSZ substrate determined by SHG polarimetry.

Then, the nonlinear optical response of the thin film was investigated. By irradiating the YbFe_2_O_4_ film with an 800 nm pulse, a finite signal of 400 nm pulse was observed, as shown by red circles in Figure 4a. (Both the polarizations of the incident 800 nm pulse and the detected 400 nm pulse are parallel to YSZ [11$\bar{2}$] axis.) A fitting analysis of the obtained data, assuming that the signal is proportional to the square of the incident intensity, is shown by the solid black line in Figure 4a. This result indicates that the film exhibits an SHG signal and has a polar structure with broken inversion symmetry.

To further investigate SHG in YbFe_2_O_4_ film, the azimuth angle dependence of SHG was investigated. Red and blue circles in Figure 4b,c show the angular dependence of the SHG intensity polarized along the YSZ [11$\bar{2}$] axis and the [1$\bar{1}$0] axis, respectively. (See also Figure 3a.) The numbers on the circumference denote the angle between the polarization of the incident laser light (800 nm) and the [11$\bar{2}$] direction of the substrate. There is a significant SHG in both polarizations, and the angular profiles of four clover-shaped leaves are observed, almost identical to that of a single bulk crystal [9].

In general, the SHG intensity (*I*_SH_) is related to the incident optical electric field (*E*) using the equation, *I*_SH_ $\propto \;\left|P^{\left(2\right)}\right|$^2^$\propto \left|\varepsilon_{0}\chi^{\left(2\right)}EE\right|$^2^, where χ^(2)^ is the second-order nonlinear susceptibility and ε_0_ is the dielectric constant in a vacuum. We assumed that the thin film crystal has the monoclinic *C_m_* symmetry (Exactly speaking, we can consider three different point groups to reproduce the observed SHG profile in Figure 4b,c; i.e., 1 in triclinic, *C*_m_ in monoclinic, and mm2 in orthorhombic. However, there is no reason of triclinic structure from XRD in the films investigated. In addition, the crystal analysis based on neutron diffraction measurement for a single crystal of YbFe_2_O_4_ [9], indicates the symmetry should be monoclinic as long as the charge ordering of iron ions are involved. Thus, we chose *C*_m_ among the three) and defined the substrate’s [11$\bar{2}$] (0°) and [1$\bar{1}$0] (90°) directions as *a*_m_ and *b*_m_ in the assumed monoclinic lattice, respectively. Then, the relationship between SHG intensity and *θ*, an angle between the polarization direction of the incident pulse and YSZ [11$\bar{2}$], is described as follows [24]:

(1) $$I_{\mathrm{SH}}{}^{\left(a\right)}\propto {\;\varepsilon }_{0}{}^{2}E^{4}{\left(\;d_{11}\cos^{2}\theta +d_{12}\sin^{2}\theta \right)}^{2}$$

(2) $$I_{\mathrm{SH}}{}^{\left(b\right)}\propto {\;\varepsilon }_{0}{}^{2}E^{4}{\left(d_{26}\sin2\theta \right)}^{2}$$

where $I_{\mathrm{SH}}{}^{\left(a\right)}$ and $I_{\mathrm{SH}}{}^{\left(b\right)}$ denotes the SHG component observed along *a*_m_ and *b*_m_ axis, respectively, assuming the monoclinic *C_m_* symmetry. The black lines in Figure 4b,c are the results of fitting the SHG angle profiles based on Equations (1) and (2) and agree with the experimental results. These fitting results show that the YbFe_2_O_4_ thin film deposited on YSZ substrates is monoclinic *C_m_*, whose *b*_m_ and *a*_m_ axes correspond to the substrate’s [$1\bar{1}0$] and [11$\bar{2}$] directions, respectively. The ratio of the obtained contracted tensor components is *d*_11_: *d*_12_: *d*_26_ = 1: –1.1:1.5, which almost corresponds to that in the single crystal [9].

Figure 4d summarizes the relationship between the crystal structure of YbFe_2_O_4_ thin film and the axes of the YSZ substrate. The polarization structure is described based on the *C_m_* point group, which is identical to that in a single crystal. What should be noticed is that the direction of the crystal axes is uniquely determined by that of the YSZ substrate, indicating that the *W*-layer of the YbFe_2_O_4_ film grows in a hexagonal lattice, reflecting the atomic arrangement of the YSZ substrate.

Finally, terahertz radiation from the film of YbFe_2_O_4_ was investigated. In a crystal with broken inversion symmetry, difference frequency generation (DFG), one of the second-order nonlinear optical effects, is observed in addition to SHG. Since the femtosecond pulses used in the experiment have a finite bandwidth (Full width at half maximum≈50 nm), DFG within the incident pulse is expected to occur in a crystal without inversion symmetry, generating terahertz waves [25]. (See Figure 3b for the experimental setup).

**Figure 5:**
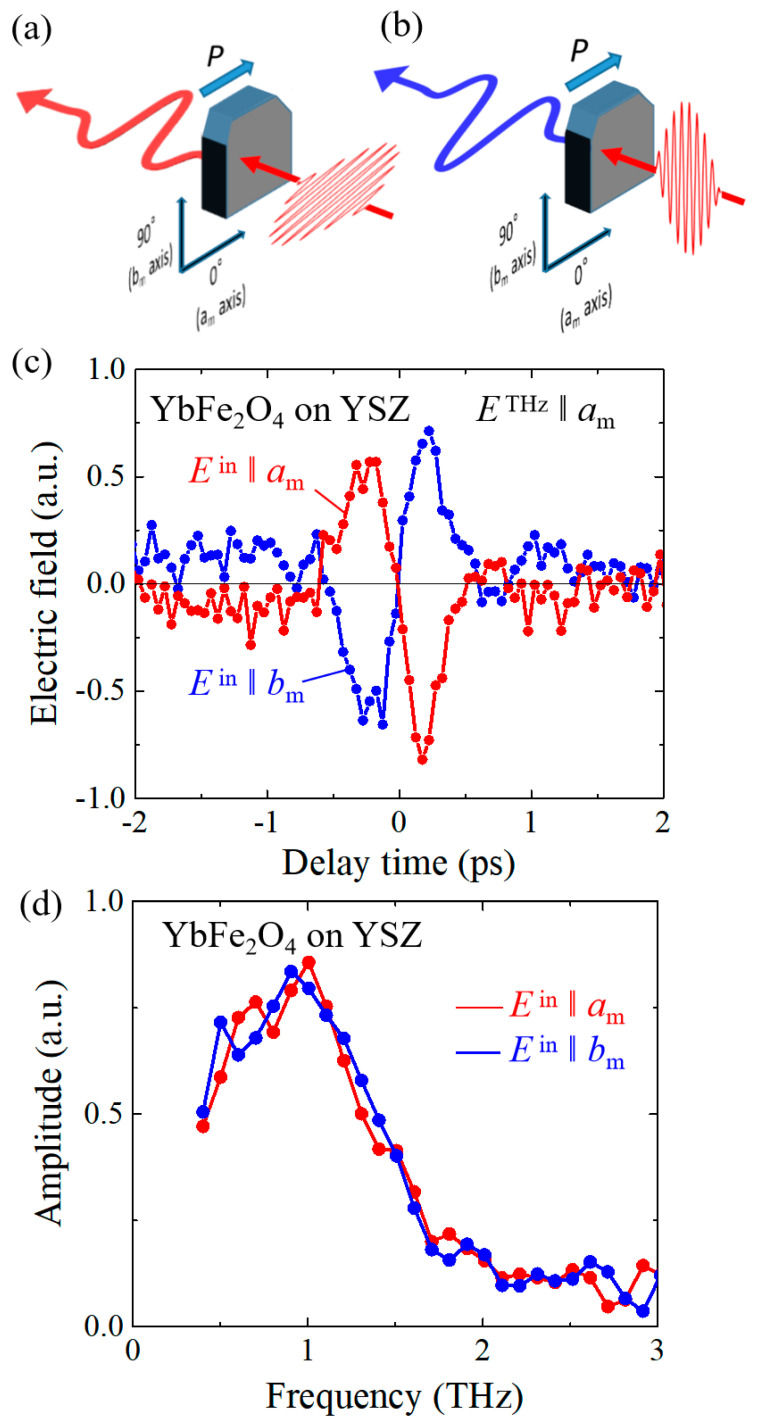
(**a**,**b**) Schematics to illustrate the relationship of the polarization of the 800 nm pulse and that of generated terahertz pulse. (**c**) Waveforms of the terahertz pulse emitted in the direction of *a_m_* axis from YbFe_2_O_4_ thin film. The red (blue) circles denote the terahertz pulse generated when the polarization of the incident fundamental pulse (*E*_in_) is set along the *a_m_* (*b_m_*) axis. (**d**) Amplitude spectra calculated by Fourier transform analysis from the waveforms in Figure 5c.

In Figure 5c, we plotted waveforms of terahertz pulse polarized along the *a*_m_ axis, which are generated when the polarization of the fundamental pulse is along the *a*_m_ axis ($E_{\mathrm{THz}}{}^{\left(a\right)}$, red circles) and *b*_m_ axis ($E_{\mathrm{THz}}{}^{\left(b\right)}$, blue circles). The relationships between the polarization of the incident 800 nm pulse and the generated terahertz pulse in the measurement are schematically depicted in Figure 5a,b. Despite the small thickness of approximately 50 nm, we could observe clear terahertz radiation signals in both polarizations, supporting the polar structure of the YbFe_2_O_4_ film confirmed by the SHG polarimetry. It is important to notice that the shapes of the two profiles of $E_{\mathrm{THz}}{}^{\left(a\right)}$ and $E_{\mathrm{THz}}{}^{\left(b\right)}$ are symmetrical about the horizontal axis and their signs are opposite to each other at all the delay times. In the *C_m_* symmetry, $E_{\mathrm{THz}}{}^{\left(a\right)}\propto d_{11}^{\mathrm{THz}}\varepsilon_{0}E^{2}$ and $E_{\mathrm{THz}}{}^{\left(b\right)}\propto d_{12}^{\mathrm{THz}}\varepsilon_{0}E^{2}$ [26] as described in Equations (1) and (2), indicating that the absolute value of $d_{11}^{\mathrm{THz}}$ is quite similar to that of $d_{12}^{\mathrm{THz}}$ but their signs are opposite. This result is consistent with the conclusion of SHG polarimetry that $\frac{d_{12}}{\;d_{11}}\approx -1.1$ at the wavelength of 800 nm.

Figure 5d shows the Fourier amplitude spectra calculated from the terahertz waveforms obtained by irradiating the fundamental pulse polarized along the *a_m_* axis (red circles) and the *b_m_* axis (blue circles). The calculated amplitude spectra are almost identical, reflecting the symmetric time profiles in both polarizations. The emitted spectra formed a broad peak at approximately 1 THz and covered the photon energy region up to approximately 2 THz.

To estimate the magnitude of the terahertz electric field emitted from the YbFe_2_O_4_ film, we also measured the terahertz pulse from a ZnTe, a typical nonlinear crystal usually used for terahertz wave generation, using the same experimental configuration for the YbFe_2_O_4_ film, as depicted in Figure 3b. (The thickness of the ZnTe crystal investigated is 1 mm.) The maximum value of the electric field radiated from the bulk ZnTe, whose waveform is shown in Supplementary Figure S1, is approximately 625 times as large as that observed in the YbFe_2_O_4_ film [27]. If we assume that the electric field of the generated pulse is proportional to the thickness of the film and the intensity of the irradiated 800 nm pulse, we can estimate that the magnitude of the terahertz field from YbFe_2_O_4_ corresponds to approximately 9.2% of that in ZnTe. This result suggests that YbFe_2_O_4_ has a large χ^(2)^ and has a potential application as a future nonlinear optical device.

It is noteworthy that the shape of the generated terahertz waveform is determined by the relative relationship between the polarization of the ferroelectric material and the incident pulse. It is generally difficult to invert the terahertz waveform without rotating the ferroelectric material or nonlinear crystal in real space. However, with YbFe_2_O_4_, the generated terahertz pulse can easily be inverted by rotating an 800 nm incident pulse by 90°, as shown in Figure 5a,b. This result from the asymmetric relationship of $d_{11}^{\mathrm{THz}}$ and $d_{12}^{\mathrm{THz}}$ implies that YbFe_2_O_4_ can be applied as a terahertz wave generator in which the direction of the generated electric field can be easily switched.

## 4. Conclusions

In summary, we fabricated YbFe_2_O_4_ thin film on YSZ (111) substrates using a magneto-sputtering technique and investigated two types of second-order nonlinear effects, SHG, and terahertz radiation. The nonlinear phenomena directly reveal a polar structure of the fabricated thin film crystal. The azimuth angle dependence of the SHG profiles for the YbFe_2_O_4_ film clarifies the point group of the thin film crystal (monoclinic *C_m_*) and the relationship between the direction of the polarization in the crystal and the crystal axes of the YSZ substrate. The magnitude of the generated terahertz electric field was found to be as large as 9.2% of that of ZnTe, a typical nonlinear optical device. This study demonstrates the epitaxial growth of YbFe_2_O_4_ thin films with polar symmetry similar to that of bulk crystal and provides the way for their nonlinear optical device applications of electronic ferroelectrics.

## Figures and Tables

**Table 1:** Experimental conditions for sputtering and the structural parameters of the fabricated YbFe_2_O_4_ thin film.

O_2_ Partial Pressure (10^−6^ Pa)	Retention Time (min)	*c*-axis Lattice Constant (Å)	Film Thickness (nm)
1.36	8	25.125(2)	~50

## Data Availability

Data availble on request.

## Supplementary Materials

The following supporting information can be downloaded at: https://www.mdpi.com/article/10.3390/ma16051989/s1. See supplementary materials for the waveform of the electric field radiated from the bulk ZnTe in our measurement system in Figure 3b.
