# Supplementary figures and images for: Nonlinear Optical Properties in an Epitaxial YbFe2O4 Film Probed by Second Harmonic and Terahertz Generation
Source: Materials (Basel). 2023 Feb 28;16(5):1989. doi: 10.3390/ma16051989 (PMC10004718; doi:10.3390/ma16051989)

## Supplementary Materials

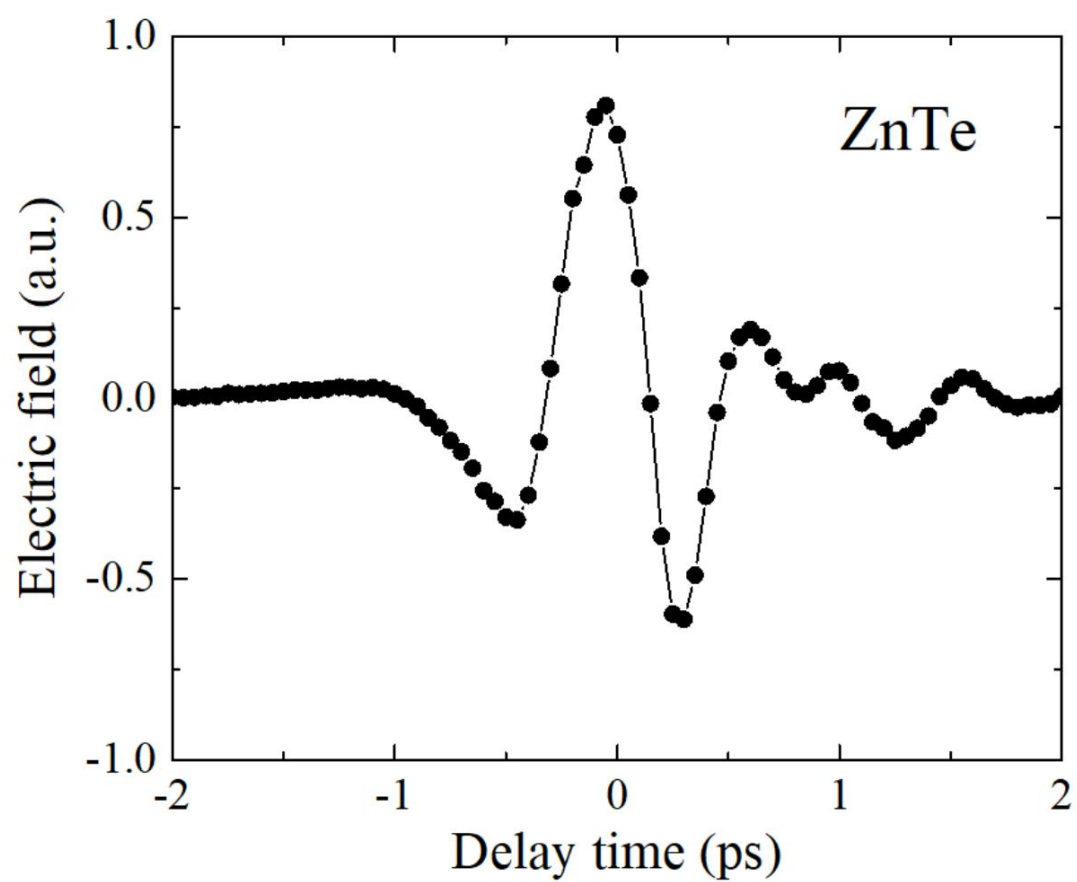

Figure S1: Waveform of the electric field radiated from the bulk ZnTe.

Supplement: Supplementary file 1 [file materials-16-01989-s001.zip › materials-2239104-supplementary.pdf]
